# Evolutionary and Structural Insights into the RNA Polymerase I A34 Protein Family: A Focus on Intrinsic Disorder and Phase Separation

**DOI:** 10.3390/genes16010061

**Published:** 2025-01-07

**Authors:** Bruce A. Knutson, Lawrence I. Rothblum

**Affiliations:** 1 Department of Biochemistry and Molecular Biology, SUNY Upstate Medical University, Syracuse, NY 13210, USA; 2 Department of Cell Biology, University of Oklahoma Health Sciences Center, Oklahoma City, OK 73104, USA

**Keywords:** A34.5, A49, PAF49, PAF53, Pol I transcription, liquid–liquid phase separation

## Abstract

Background: Eukaryotic RNA polymerase I consists of 12 or 11 core subunits and three dissociable subunits, Rrn3, A34, and A49. The A34 and A49 subunits exist as a heterodimer. In silico analysis of the A34 family of transcription factors demonstrates a commonly shared domain structure despite a lack of sequence conservation, as well as N–terminal and C-terminal disordered regions. The common structure of A34 has an N–terminal disordered region followed by a dimerization domain that, in conjunction with A49, contributes to a fold that resembles the TFIIF core. This in turn is followed by a short region that cryo-EM demonstrates resembles an arm and intimately interacts with the PolR1A, PolR1B, and PolR1C subunits of Pol I. Analyses: This Pol I–binding domain is then followed by a region that is not resolved in cryo-EM and is predicted to be intrinsically disordered. Interestingly, the size/length of this disordered structure increases from yeast to humans, and is composed of repeats with unique sequence and biochemical features that also increase in number. Further analyses of the A34 CTD (carboxy–terminal domain) indicate that it has a high probability of undergoing liquid–liquid phase separation. Conclusions: We suggest that this intrinsically disordered domain found in the A34 family of Pol I transcription factors serves a function similar to the CTD of the PolR2A subunit in coordinating transcription initiation and elongation and RNA processing. Lastly, we propose that dynamic acetylation of PAF49 may regulate interactions of the intrinsically disordered CTD and thereby specify liquid–liquid phase separations. Overall, we propose a new paradigm for a repeat-containing CTD in Pol I transcription.

## 1. Introduction

RNA polymerase I (Pol I) is responsible for the synthesis of three of the four ribosomal RNAs (rRNAs), which constitute approximately 80% of the cellular RNA. Pol I is a highly specialized enzyme complex that includes three polymerase-associated factors, Rrn3, and a heterodimer of A34 and A49 [1]. The structure of mammalian Pol I is similar to its yeast counterpart, with the notable exception that mammalian Pol I lacks the core subunit RPA14 [2,3]. The PAF49 subunit of human Pol I (encoded by the *POLR1E* gene), and its yeast homolog A34, form a heterodimer with another Pol I subunit, PAF53 and A49, respectively, creating a dissociable subcomplex within the Pol I enzyme [4,5,6].

Structurally, the dimerization of PAF49/A34 and PAF53/A49 is mediated by their N-terminal beta barrel domains, which bind the lobe domain on the outer surface of the Rpa2 subunit [7,8,9]. The PAF49/53 heterodimer and its yeast ortholog play several roles throughout the Pol I transcription process during the stages of initiation, promoter opening, and elongation [10]. One of the most notable features of PAF49/A34 is its long-extended C-terminal arm domain, which lacks any tertiary structure [8,9]. In human PAF49, only a small segment of this “arm,” adjacent to the dimerization domain, has been resolved in current high-resolution structures, where it hugs the Pol I core to aid the heterodimer’s interaction with Pol I lobe domain [2,3]. The structure of the remainder of the C-terminal arm remains unresolved.

Interestingly, the yeast subunit, A34, is not essential for rDNA transcription [11]. In addition, deletion of the yeast heterodimer affects the binding and release of a third subunit (Rrn3) from the yeast holo Pol I and reduces the rate of cell division [12]. In contrast, its mammalian homolog is essential for rDNA transcription and cell growth [13]. Further, Panov et al. have reported evidence for the growth-regulated tyrosine phosphorylation of hPAF49 in initiation-competent Pol I complexes in HeLa cells and showed that hPAF49 can interact with the mammalian rDNA transcription factors UBF and SL1 [14,15].

In this bioinformatics study, we utilized various bioinformatic and computational tools to analyze the C-terminal domain (CTD) of the A34 protein family across species. We discovered that the CTD of A34 is evolutionarily conserved across species yet variable in length. We also found that as the A34 CTD increases in size and complexity from yeast to human through an increase in the number of repeated units, yet they retain their intrinsically disordered nature and well as similar sequence features. Finally, various predictions reveal that the CTD is intrinsically disordered and has a high propensity for liquid–liquid phase separation (LLPS). LLPS is a process where proteins demix from the surrounding solution to form concentrated, dynamic assemblies without a defined membrane, which plays a critical role in organizing cellular biochemistry and regulating biological processes [16,17]. The finding that the propensity of the CTD of A34 to undergo LLPS is an evolutionarily conserved feature suggests that it may provide a platform to modulate Pol I transcriptional activity and coordinate the interactions of Pol I with additional transcriptional regulatory factors and possibly rRNA processing factors in a manner reminiscent of the Pol II CTD.

## 2. Materials and Methods

### 2.1. Prediction of Intrinsic Disorder

To analyze the intrinsic disorder propensity of the A34 protein, we utilized several programs with default settings. PONDR (Predictor of Natural Disordered Regions; http://www.pondr.com/, accessed on 1 November 2024) (Molecular Kinetics, Inc., Indianapolis, IN, USA), IUPred2A (https://iupred2a.elte.hu/, accessed on 1 November 2024) [18,19], and GlobPlot (http://globplot.embl.de/, accessed on 1 November 2024) were employed to identify disordered regions in the protein sequence [20]. Additionally, PrDOS (https://prdos.hgc.jp/cgi-bin/top.cgi, accessed on 1 November 2024) provided predictions of disordered regions based on a combination of structural and sequence features [21].

### 2.2. Protein Structure and Function Prediction

To predict structural features and assess functional attributes of the A34 protein, we used Robetta (https://robetta.bakerlab.org/, accessed on 1 November 2024) for structural modeling using default settings [22] and AlphaFold 2 (https://alphafold.ebi.ac.uk/, accessed on 1 November 2024) for high-accuracy three-dimensional structure predictions [23].

### 2.3. Evolutionary Conservation Analysis

Evolutionary conservation of A34 protein sequences was assessed using ETE Toolkit (http://etetoolkit.org/, accessed on 1 November 2024) for phylogenetic tree analysis and visualization [24], and Aminode (http://www.aminode.org/search, accessed on 1 November 2024) to identify conserved regions critical for protein function [25]. Both programs were run with default parameters to highlight conserved domains across species.

### 2.4. Sequence Alignment and Comparison

Sequence alignments and comparative analyses were conducted using EMBOSS Dotmatcher (https://www.ebi.ac.uk/jdispatcher/seqstats/emboss_dotmatcher, accessed on 1 November 2024) to generate dot plots for visualizing sequence similarity [26]. Additionally, FELLS (http://old.protein.bio.unipd.it/fells/, accessed on 1 November 2024) was utilized to align sequences and provide functional insights [27]. Additionally, GPS-PAIL (http://pail.biocuckoo.org/, accessed on 1 November 2024) was used to identify acetylation post-translational modification sites within the sequences [28] Default settings were applied for both programs.

### 2.5. Sequence Logo and Motif Visualization

To visualize conserved motifs within the A34 protein sequences, WebLogo (https://weblogo.berkeley.edu/logo.cgi, accessed on 1 November 2024) was used [29]. Sequence logos were generated to highlight conserved regions across aligned sequences, and the program was run using default settings.

### 2.6. Phase Separation and Aggregation Propensity

The potential for phase separation and aggregation of A34 was evaluated using catGRANULE (http://s.tartaglialab.com/update_submission/876957/cdb5fa503d, accessed on 1 November 2024) to identify aggregation-prone regions [30]. FuzDrop (https://fuzdrop.bio.unipd.it/predictor, accessed on 1 November 2024) was used to predict regions involved in liquid–liquid phase separation [31], while PSPredictor (http://www.pkumdl.cn:8000/PSPredictor/, accessed on 1 November 2024) assessed phase separation propensity using sequence-based features [32]. Additionally, PhaSePred (http://predict.phasep.pro/, accessed on 1 November 2024) was used to predict phase-separating regions within the A34 protein sequence [33]. These programs were run with default settings.

## 3. Results and Discussion

### 3.1. Structural Similarities and Evolutionary Trends in the A34 CTD

**Figure 1:**
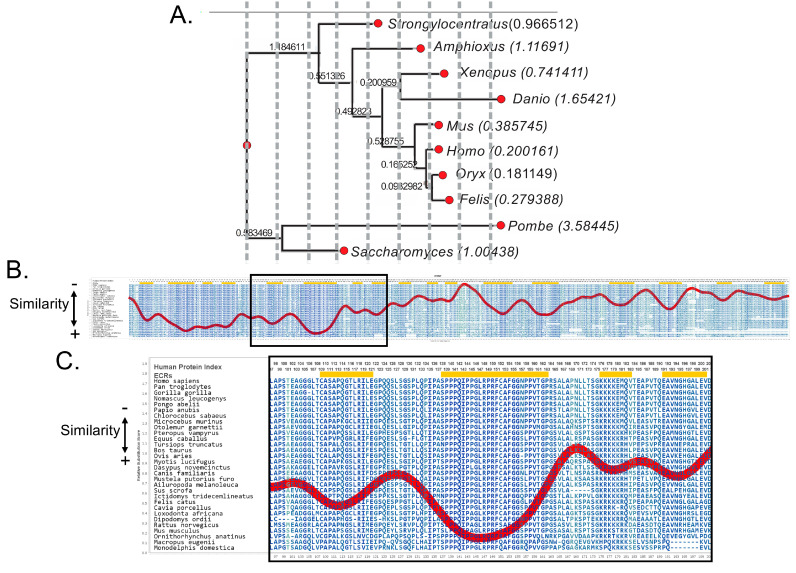
Analysis of the evolutionary conservation, or lack thereof, of A34. (**A**) Abbreviated phylogenetic tree of the A34 family members generated by phylogenetic analysis pipeline by ETE3. Branch lengths values are shown to the left of the genus name for each A34 protein analyzed, where longer branch lengths indicate more evolutionary change. (**B**) Aminode analysis of A34. Local maxima represented by the red line indicate protein regions with relatively low evolutionary constraints, while minima indicate evolutionarily constrained regions (ECRs) [25]. The region enclosed in the black rectangle represents the dimerization domain and the defined Pol I-binding domain of A34. (**C**) A close−up of the most conserved portion of A34. It has been hypothesized that this region represents the core of the Pol I-binding domain of A34 [13].

In our study, we aligned the sequences of the A34 protein from 10 species, ranging from yeast to humans, and constructed a phylogenetic tree to illustrate their evolutionary relationships (Figure 1A, Table 1) [24]. The analysis reveals a gradient of conservation across different taxa. In yeast species like *Saccharomyces* and *Schizosaccharomyces pombe*, A34 shows significant divergence, indicated by longer branch lengths, suggesting substantial evolutionary changes. In contrast, organisms like *Xenopus* and *Danio* (zebrafish) exhibit moderate conservation, while in mammals, including *Mus* (mouse), *Felis*, and *Homo sapiens* (human), A34 is highly conserved.

The Aminode analysis of vertebrate A34 family members reveals a relatively low level of sequence variation within the first 166 amino acids [25] (Figure 1B). This conserved region, boxed in the figure, is particularly significant because it contains the dimerization domains essential for the interaction between A34 and A49. Beyond this point, the sequence diverges into the C-terminal arm domain, which is noted for its more variable and flexible structure. Despite the low sequence identity—less than 30%—between the A34 orthologs, the structural similarities within the conserved N-terminal region are striking. Included in this conserved domain is the region of A34 that facilitates its interaction with the lobe of Pol I (Figure 1C). This suggests that the conservation of key structural elements, especially the dimerization domain, may contribute to “static” functions, while the C-terminal arm domain likely contributes to the dynamic functions of the complex.

**Figure 2:**
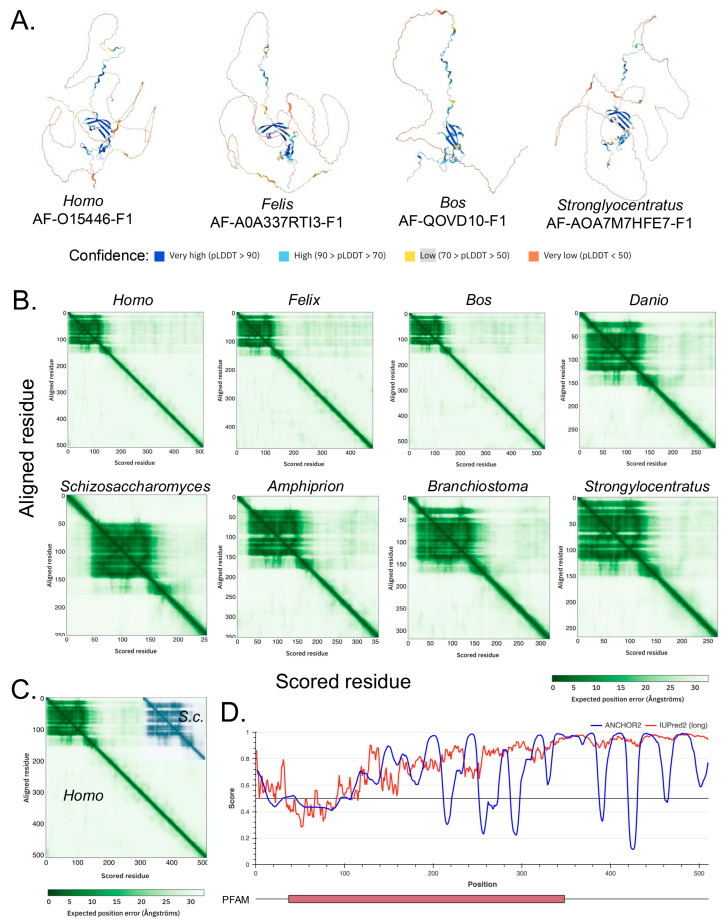
AlphaFold-predicted structures for various A34 homolog and predicted alignment error (PAE) graphs. (**A**). AlphaFold-derived structures for indicated A34 homologs. Confidence of structure predictions is indicated by the colors below. (**B**) PAE graphs for the A34 homologs. The white areas of the graphs correlate with regions of low confidence in domain positioning. Note that the graphs contain regions of high confidence circa the N-termini and regions of low confidence extending to the C-termini. (**C**) Superposition of the PAE graphs for human and yeast (*S.c*.) A34. The two graphs have been superposed for the first 200 residues of each protein. (**D**). IUPRED2A analysis of human A34 predicts that the C-terminus will be intrinsically disordered [18,19]. IUPred2 and ANCHOR2 scores are shown in red and blue. Below the graph is a schematic of human A34 protein, with the red box denoting the conserved PFAM domain.

The derived and predicted structures for the A34 orthologs are also similar, up to a point (Figure 2A). None of the derived, i.e., cryo-EM, structures, e.g., PDB5W66 [34], contain amino acids close to the C-termini of the respective A34 orthologs (Figure 2A). The regions derived in the cryo-EM structures coincide with the blue in the AlphaFold 2 predictions (structures with either confidence or high confidence) [35]. In contrast, the regions not otherwise contained in the cryo-EM data appear as amber/brown in the AlphaFold models, i.e., their structures are predicted with low or very low confidence (amber/brown). The predicted alignment errors for the A34 homologs indicate ordered, N-terminal regions of relatively similar lengths, approximately 166 aa, the size of which is relatively conserved across species. This region is followed by a short, disordered segment in *Saccharomyces cerevisiae* A34 and progressively longer disordered regions in other homologs (Figure 2B). Furthermore, as illustrated in Figure 2C, the lengths of these disordered regions increase progressively from yeast (~84 aa) to humans (344 aa); with the vertebrate species such as humans and *Oryx* having the longest, most disordered CTD domains. This trend suggests a potential correlation between the complexity of the organism and the length of the disordered CTD. The expansion of the disordered CTD in higher organisms, particularly vertebrates, could imply an increasing complexity in the regulation and interaction of RNA polymerase I with other cellular components. The intrinsically disordered structures at the C-termini are also predicted by IUPred2A [19] (Figure 2D).

The presence of a disordered region at the C-terminus of A34 (A34-CTD), as well as the observed increase in the size of this disordered domain from yeast to humans, is reminiscent of the changes observed in the C-terminal domain (CTD) of the highest-molecular-weight subunit of RNA polymerase II (Table 2) [36]. The paralogous RNA polymerase I subunit (Pol I) lacks a CTD of its own. In yeast, the CTD of Rpb1 contains 26 copies of a consensus heptad repeat, while in mammals, the CTD contains 52 copies of the repeat [37]. This expansion in the number of repeats in higher eukaryotes correlates with the increased complexity of transcriptional regulation required in these organisms. Similarly, the elongation of the A34 CTD across species might suggest a parallel evolutionary trajectory, where the increase in the size and flexibility of this disordered domain supports more sophisticated regulatory functions. This evolutionary trend highlights the functional importance of the disordered A34 CTD.

Further, extrapolating from the available cryo-EM data, it appears that the approximate locations of the Pol II CTD and the A34 CTD within their respective polymerase structures are strikingly similar. Both CTDs are situated opposite the cleft and/or near the RNA exit channels of their respective polymerases. This strategic positioning is significant, as it suggests a functional analogy between these domains across different RNA polymerases. The proximity of the CTDs to the RNA exit channels implies a potential role in modulating the exit of nascent RNA transcripts, possibly through interactions with various transcriptional regulators or RNA processing factors.

The location of the CTD near the RNA exit channel is well established in Pol II, where the CTD plays a crucial role in coordinating recruitment and the transition from transcription initiation to elongation, as well as in recruiting factors involved in RNA processing and termination [38,39,40]. By analogy, the similar placement of the A34 CTD in RNA polymerase I suggests that it may also be involved in coordinating critical post-initiation events during rRNA synthesis. This could include the recruitment of ribosome biogenesis factors or the regulation of transcriptional pausing and termination.

### 3.2. Repeated Sequences in the A34 CTD

**Figure 3:**
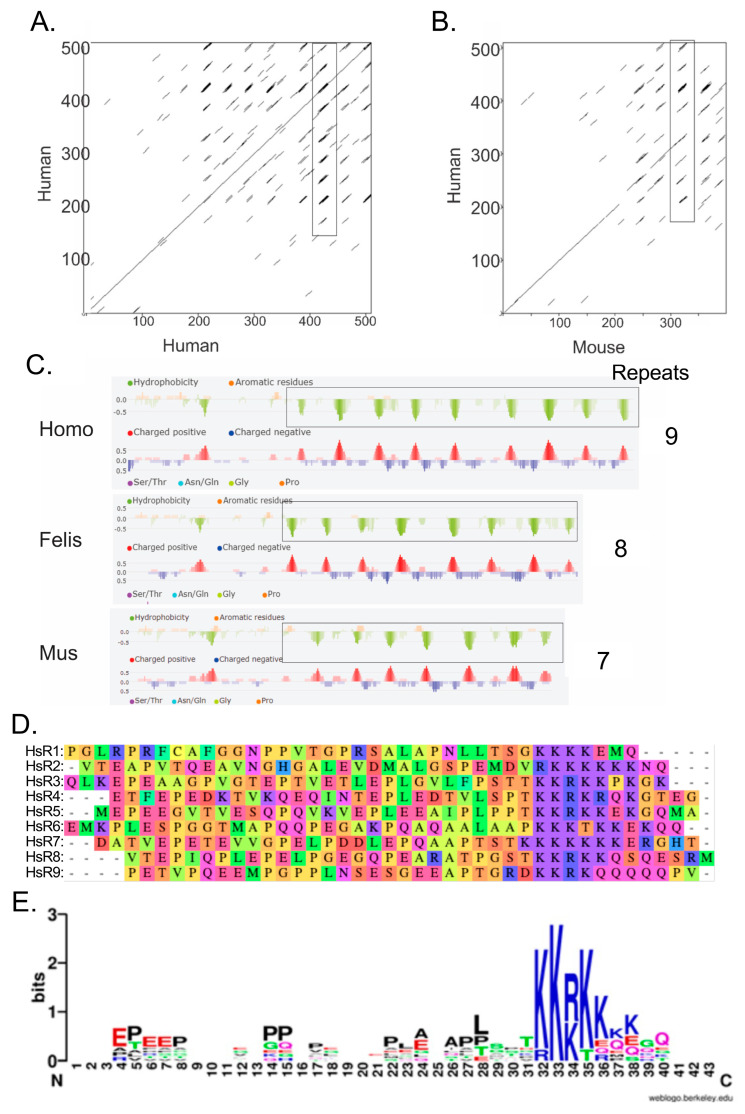
Identification of repeats in the CTD of human A34. (**A**) Dotmatcher dot plot of human A34 vs human A34. A stretch of repeated sequences is enclosed in a rectangle. (**B**) Dot plot of human A34 vs mouse A34 illustrating the conservation of the repeated elements. A stretch of repeated sequences found in both human and mouse A34 is enclosed in a rectangle. (**C**) Portions of the FELLS [28] analyses of the human, cat, and mouse A34 homologs. (**D**) Alignment of human A34 CTD repeats. (**E**) WebLogo display of the consensus sequence for the nine repeats found in the CTD of human A34 [29]. All analyses were carried out with default settings.

We considered that, like the Pol II CTD, the A34 CTD may also consist of repeated sequences that play a critical role in its function. Alignment of the sequence of human A34 with itself using Dotmatcher demonstrated repeated sequences (additional horizontal lines in the dot plot in Figure 3A). (*NB:* Alignment of a sequence against itself should demonstrate a straight line on the diagonal with a slope of 45 degrees. The presence of additional diagonal lines is indicative of repeated sequences.) Similarly, repeated sequences were apparent in the Dotmatcher plot of human A34 versus mouse A34 (Figure 3B).

Examination of the hydropathy and charge plot of human, cat, and mouse A34 homologs (Figure 3C) revealed intriguing features in the CTD. The presence of recurring negative peaks and hydrophobic clusters again suggested the presence of repeated sequences. These regions rich in acidic residues and proline, characterized by negative charge, are followed by clusters of positively charged lysine residues. The number of lysines within these clusters varies significantly, ranging from just a few to more than five on average. Specifically, analyses using Dotmatcher and FELLS [26,27] confirmed the presence of repeated sequences within the A34 CTD (Figure 3A–C). A WebLogo analysis of all the aligned human A34 CTD repeats clearly reveals a distinctive pattern: a lysine cluster is consistently preceded by a long stretch of acidic residues interspersed with regularly spaced proline residues (Figure 3D,E) [29].

Proline residues are particularly abundant in disordered proteins and play a significant role in shaping the conformational dynamics of these regions [41,42,43]. In the case of the A34 CTD, the regular spacing of prolines likely increases the probability that the domain adopts a more open and extended conformation rather than a compact one. This extended state could have important implications for potential protein–protein interactions, as it may facilitate the accessibility of interaction sites within the CTD. Moreover, prolines are known to undergo cis-trans isomerization, a process that can further influence the conformational states of the CTD [44,45]. The WebLogo analysis also highlights a moderate frequency of serine (S) and threonine (T) residues immediately upstream of the lysine clusters. These S/T residues, along with the lysine clusters themselves, are potential sites for post-translational modifications (PTMs), such as phosphorylation and acetylation. For instance, PTMs at these sites could dynamically regulate the structure and function of the A34 CTD.

Notably, the human A34 CTD contains nine such repeats. This pattern of alternating charge may facilitate interactions with various nucleic acids or proteins, contributing to the dynamic role of A34 in the nucleolus. Interestingly, the number of these repeated sequences appears to correlate with the size of the CTD, which increases progressively from yeast to humans (Table 3). As the CTD expands, so too does the number of repeats, indicating a potential evolutionary trend where the expansion of the CTD is associated with an increase in its repeat elements. This pattern suggests that the length and complexity of the CTD, along with its repeated sequences, might play a crucial role in the functional diversification of A34 across different species.

### 3.3. Assessing the Intrinsic Disorder and LLPS Propensity of the A34 CTD

The Pol II CTD has been shown to undergo liquid–liquid phase separation (LLPS), a process that can concentrate the transcriptional machinery at specific genomic loci as well as facilitate the formation of membraneless organelles [46,47]. Phase separation of the Pol II CTD plays a critical role in regulating transcriptional dynamics by coordinating the recruitment of Pol II and creating concentrated and possibly distinct hubs of transcription factors and RNA-processing enzymes. Given their parallels, we hypothesized that the A34 CTD might also possess the potential to undergo LLPS, which could similarly contribute to the regulation of rRNA transcription. To explore this possibility, we conducted several in silico analyses using established predictors of phase separation propensity. These computational tools allow us to assess whether the A34 CTD has the intrinsic properties, such as sequence composition and disorder, that are conducive to LLPS.

We first wanted to ascertain whether the entire A34 CTD is intrinsically disordered. There is no experimental structural information for much of the A34 CTD given that it has not been resolved in structural studies. Likewise, our AlphaFold analyses failed to predict a structure of the A34 CTD among all the orthologs. AlphaFold predicts these regions with low confidence, reflecting the potential intrinsic disorder of the CTD, and this agreed with IUPRED2A. To further confirm this, we used additional in silico web-based tools that include PONDR [48], GLOBPLOT2 [22], and PrDOS [23]. These tools are specifically designed to identify intrinsically disordered regions (IDRs) within proteins, offering a complementary approach to the structural predictions provided by AlphaFold.

**Figure 4:**
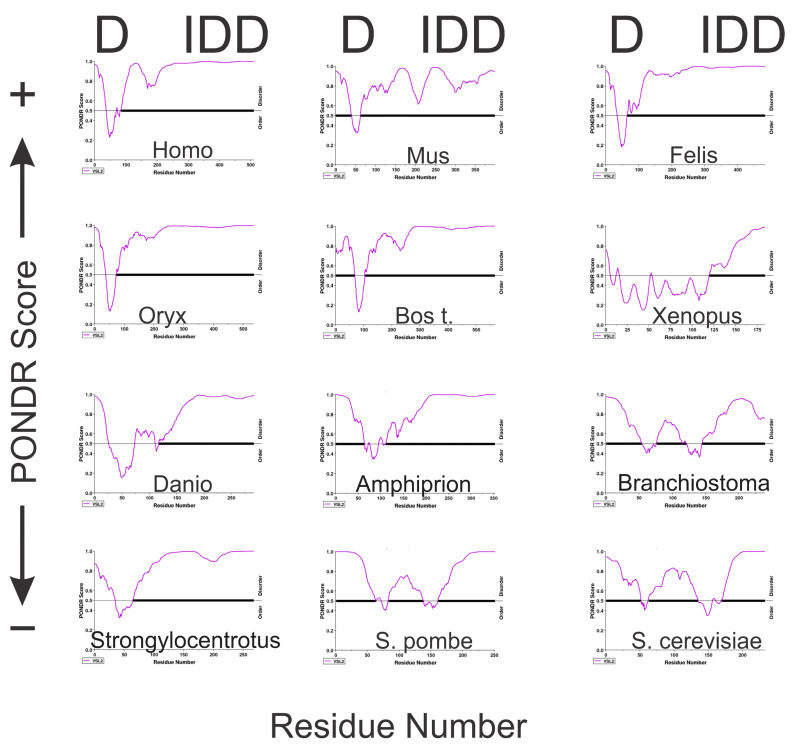
PONDR analysis of twelve eukaryotic A34 orthologs. The PONDR predictions were generated using the VSL2 algorithm (http://www.pondr.com/pondr-tut.html, accessed on 1 November 2024). A higher PONDR score is indicative of a tendency to disorder. D = dimerization domain; IDD = intrinsically disordered domain. The x axis represents the sequence residues, and the *y* axis is the PONDR score. The purple line is the PONDR predictions for each protein analyzed. The black horizontal line is the threshold between ordered (thin black line) and disordered (thick black line), with data above this line representing disorder. PONDR analyses were carried out with default settings.

**Figure 5:**
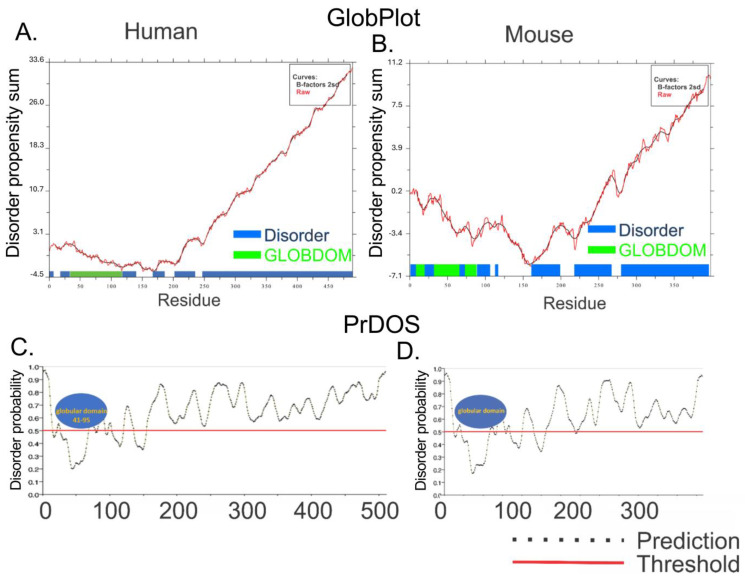
GLOBPLOT2 and PrDOS analysis of human and mouse A34. GLOBPLOT2 analysis of human (**A**) and mouse (**B**) A34 homologs. The *x* axis represents the sequence residues, and the sum of disorder propensities are on the y axis. The blue bars represent disordered residues, and the green bars correspond to the globular domain. PrDOS analysis of human (**C**) and mouse (**D**) A34 homologs. The x axis represents the sequence residues, and the y axis is the disorder probability. The dotted line indicates the prediction for each result within the protein. The red horizontal line denotes the disorder prediction probability threshold, with data above the red line indicating disorder. Globular domains are depicted as blue ovals. All analyses were carried out with default settings.

PONDR (Predictor of Naturally Disordered Regions) uses a combination of algorithms to analyze protein sequences, identifying regions with varying Ramachandran angles that correspond to disordered regions (DRs). It classifies these DRs into extended and collapsed classes, helping to discern whether a region is likely to be structured or flexible. PONDR’s analysis of the A34 family’s CTD (Figure 4) consistently predicts it as disordered, providing evidence that aligns with the low-confidence predictions from AlphaFold. GLOBPLOT2, which predicts regions of disorder by analyzing sequence profiles, similarly suggests that the CTD of A34 is unstructured or flexible (Figure 5A). This tool focuses on both local and global disorder tendencies, reinforcing the idea that the CTD does not adopt a stable conformation. Finally, PrDOS [21] (Protein Disorder Prediction System) offers a probabilistic assessment of disorder across a protein’s sequence, combining sequence-based predictions with known structural data. The PrDOS analysis (Figure 5B) also indicates a high probability of disorder in the CTD of A34. Taken together, these results strongly suggest that the CTDs of A34 are intrinsically disordered.

The nucleolus is the largest, subnuclear structure, is membraneless, and is a hub of complex biochemical activity. It is composed of some of the most intrinsically disordered proteins found within the cell [49,50] (Table 4). These proteins play a crucial role in the dynamic organization of the nucleolus by undergoing LLPS, contributing to the formation of various biological condensates [51] essential for nucleolar function. Proteins within the nucleolus often contain a mix of structured and intrinsically disordered regions (IDRs). For instance, proteins like fibrillarin have structured domains that interact with nucleic acids, while others, such as PAF49/A34, possess domains that mediate protein–protein interactions. The ability of these proteins to undergo LLPS is driven by various forces, including electrostatic interactions, hydrophobic interactions, cation–pi interactions, and pi–pi interactions, or a combination thereof. A common characteristic among proteins that participate in condensate formation is the presence of IDRs, which typically do not adopt a stable three-dimensional structure. Although tools like AlphaFold, PONDR, and ROBETTA are effective in predicting internal disorder within these proteins, additional analyses are required to assess the likelihood of condensate formation through LLPS.

**Figure 6:**
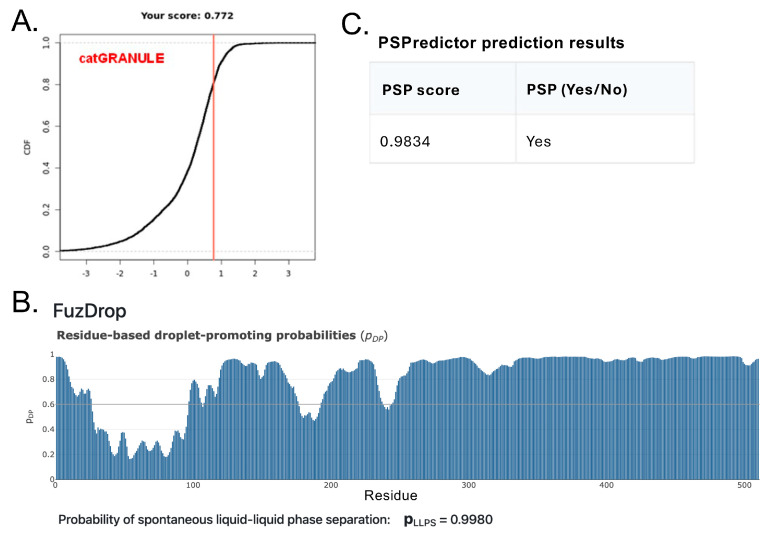
In silico predictions that the CTD of A34 will undergo LLPS. (**A**) CATgranule results display the LLPS propensity score, indicated with a vertical red line. (**B**) FuzDrop result displaying the droplet−promoting probabilities of residues (pDP). Probability for each residue is depicted with a blue bar. (**C**) PSPredicter results indicating the PSP score and overall phase separation prediction (PSP).

To explore whether the CTD of A34 is capable of undergoing LLPS, we utilized four different in silico tools, each providing unique insights into the protein’s phase separation potential (Figure 6). PSPredictor [32] is a tool designed to evaluate the likelihood that a protein can undergo phase separation by analyzing its sequence features and applying machine learning algorithms. For human A34, PSPredictor assigned a high phase separation potential (PSP) score of 0.9834, indicating a strong propensity for LLPS. Another tool, catGRANULE [30], focuses on predicting a protein’s ability to form biological granules through phase separation. It calculates a condensate-forming domain (CDF) score based on sequence attributes associated with granule formation. Human A34 received a CDF score of 0.772 from catGRANULE, further supporting the notion that it is likely to participate in LLPS. FuzDrop [52,53] is designed to identify proteins likely to undergo LLPS by assessing the presence of sequence features typical of phase-separating proteins, such as disordered regions and interaction motifs. FuzDrop provided a near-certain LLPS probability score of 0.998 for human A34, suggesting that this protein is highly likely to be involved in phase separation (Figure 6B). PSP Predictor yielded a PSP score of 0.9834, also consistent with a tendency for phase separation (Figure 6C). Finally, we employed PhaSePred [33], which integrates multiple features related to phase separation, to predict both the intrinsic phase separation potential of a protein (PS-self score) and its potential to interact with other proteins in phase-separated states (PS-partner score). Human A34 received a PS-self score of 0.889 and a PS-partner score of 0.917, further indicating its strong capacity for LLPS. The consistent results suggest that A34, like many other nucleolar components, is highly likely to undergo LLPS.

### 3.4. Post-Translational Modifications of the A34 CTD

Post-translational modifications (PTMs) play a crucial role in regulating the function of nucleolar proteins, particularly those involved in liquid–liquid phase separation. Many nucleolar proteins, such as nucleophosmin (B23) and nucleolin (C23), are heavily phosphorylated, especially in exponentially growing cells [54,55]. While direct evidence of phosphorylation on A34 is currently lacking, our research has identified that at least three of the repeats within the CTD of A34 are modified by acetylation. This acetylation has a significant impact on the functionality of A34, particularly in its ability to form a heterodimer with core Pol I [56].

Using GPS-PAIL [28], a tool for predicting acetylation sites and associated acetyltransferases, we found that CREBBP (CREB-binding protein) is the most likely acetyl-transferase responsible for modifying these sites. The acetylation status of these repeats within the CTD could modulate the interaction between A34 and Pol I, potentially influencing the overall assembly and stability of the RNA polymerase I complex.

**Figure 7:**
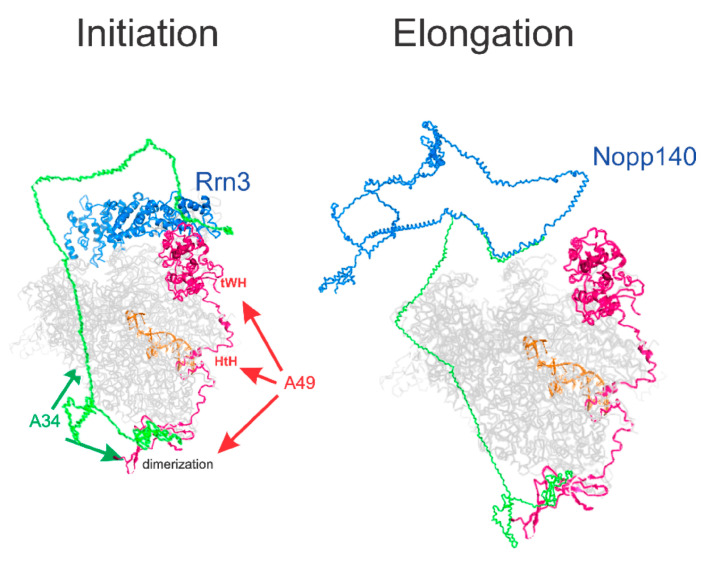
A model for the interaction of A34 with Pol I and Nopp140. The Rrn3, A49, DNA, and Pol I structures are from PDB6RQT. The core Pol I subunits are hidden. The structures of human A34 and Nopp140 were derived by Robetta (https://robetta.bakerlab.org/, accessed on 1 November 2024).

To explore this hypothesis, we utilized Robetta, a tool for protein structure prediction, to model the structure of human A34 in conjunction with the reported structure of RNA polymerase I (Figure 7). Although speculative, the resulting model aligns the known dimerization domains of A34 with those observed in existing cryo-EM images, while also proposing a potential role for the CTD in mediating further interactions with Rrn3, required for transcription initiation by Pol I [57] or other proteins involved in ribosome biogenesis, such as NOPP140 (Figure 7). This model is consistent with the findings that yeast A34 regulates the activity of yeast Rrn3 [12] but is not essential for rDNA transcription in yeast. However, mammalian PAF49 is essential for mammalian rDNA transcription. In our model, the A34/A49 heterodimer can stabilize the interaction of Rrn3 with the Pol I stalk. The yeast stalk consists of Rpa43 and Rpa14, while the mammalian Pol I stalk lacks Rpa14 [3,8,9]. It is formally possible that the yeast stalk binds Rrn3 more strongly than the mammalian stalk. Thus, additional interactions may be required to stabilize the Rrn3-Pol I complex, and this would be mediated by the mammalian A34/A49 heterodimer. Interestingly, this model places the CTD of A34 and its binding partner, NOPP140, circa the RNA exit tunnel formed by RPA1, RPA2, and the highly positively charged WH domain of RPA49 [3].

**Figure 8:**
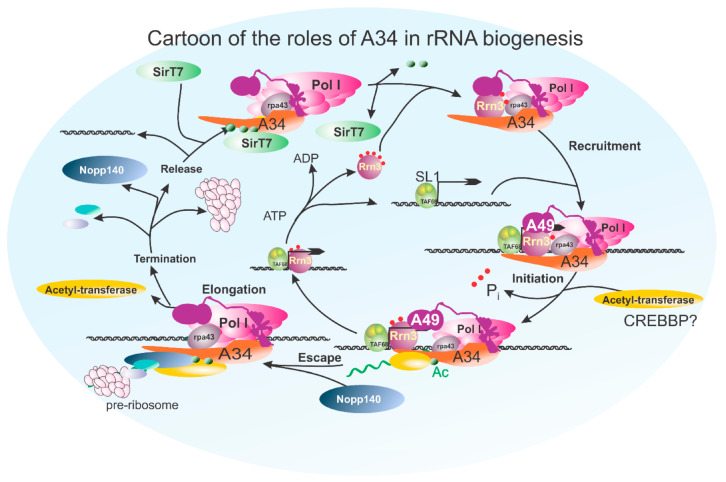
A cartoon integrating possible interactions of A34 with the RNA polymerase I transcription cycle. This figure illustrates the potential interactions and roles of the A34 subunit during the RNA polymerase I transcription cycle. A34 is depicted engaging in multiple stages, including transcription initiation, elongation, and termination. In the initiation phase, A34 facilitates recruitment or stabilization of transcription machinery at the rDNA promoter, where it is acetylated. This process occurs in conjunction with Rrn3, whose phosphorylation state regulates its association with RNA polymerase I, ensuring proper initiation complex formation. After promoter escape, A34 plays a role in maintaining polymerase stability and processivity, coordinating RNA synthesis and pre-rRNA processing through a potential interaction with both A49 and NOP140, thereby linking it directly to the pre-ribosome. Finally, in termination, the transcription complex is disassembled and recycled. During this stage, A34 is a potential target for deacetylation by SIRT7. The model integrates existing knowledge of A34’s molecular interactions and highlights its dynamic participation throughout the transcription process, potentially bridging initiation to termination events.

Additionally, we have found that SirT7, a deacetylase implicated in the regulation of ribosome biogenesis [58,59,60,61,62], interacts with A34. This interaction hints at a regulatory mechanism where SirT7 could deacetylate A34, potentially increasing the proportion of Pol I that associates with the A34/A49 heterodimer. Such a deacetylation event might enhance ribosome biogenesis by promoting a more stable and active Pol I complex, thereby facilitating efficient rRNA transcription. Figure 8 is a cartoon predicting the pos-translational modification(s) of A34 and summarizing the putative roles of A34 in rDNA transcription and pre-rRNA processing in coordination with the regulation of Rrn3 during the Pol I transcription cycle.

The potential for post-translational modifications, such as acetylation, to regulate the CTD of A34 intersects with its capacity for LLPS. Acetylation of lysine residues within the CTD could influence its phase separation behavior by altering the charge distribution and interaction dynamics of the region. This modification may either promote or inhibit LLPS, depending on the cellular context and the specific interactions involved. Given the role of liquid–liquid phase separation in organizing the nucleolar architecture, it is plausible that acetylation could serve as a switch that modulates the phase separation properties of A34, thereby fine-tuning its involvement in nucleolar functions.

## 4. Conclusions

In conclusion, our study provides insights into the structural and functional evolution of the A34/PAF49 subunit within RNA polymerase I across species, with a focus on its extended, intrinsically disordered C-terminal domain (CTD). The conserved propensity of the CTD for liquid–liquid phase separation and the presence of repeated sequences suggest it may facilitate dynamic interactions in the nucleolus, akin to the regulatory role of the Pol II CTD. Furthermore, the potential for post-translational modifications, such as acetylation, highlights a regulatory layer that could modulate the structural conformation and interaction capabilities of A34, supporting its role in coordinating transcriptional and ribosome biogenesis activities (Figure 8). Our findings underscore the evolutionary adaptation of A34, suggesting that its CTD may be crucial for fine-tuning Pol I transcription and nucleolar organization in complex eukaryotic systems.

## Figures and Tables

**Table 1:** Gene/protein names.

Gene	Protein	Aliases
RPA34/POLR1G	DNA-directed RNA polymerase I subunit RPA34	A34, A34.5, PAF49, RNA polymerase I-associated factor PAF49, Antisense to ERCC-1 protein (ASE1), CD3-epsilon-associated protein (CD3EAP)
RPA49/POLR1E	DNA-directed RNA polymerase I subunit RPA49	A49, PAF53, DNA-directed RNA polymerase I subunit E, RNA polymerase I-associated factor 1, RNA polymerase I-associated factor 53

**Table 2:** Amino acid length of the Pol II and A34 CTDs.

Organism	Pol II CTD Length	A34 CTD Length
Yeast	~182 amino acids	80 amino acids
Human	~470 amino acids	350 amino acids

**Table 3:** Number of A34 CTD repeats in various species.

Organism	Repeat Number
*Homo s.*	9
*Felix c.*	8
*Mus m.*	7
*Danio r.*	4
*Strongylocentratus p.*	3
*Drosophila m.*	3
*Schizzosacharomyces p.*	3
*Saccharomyces c.*	3

**Table 4:** Precent disorder of various human nucleolar proteins.

Nucleolar Protein	Percent Disordered
NOPP140	91
PAF49	66.8
Nucleolin (C23)	64.9
GAR1	59.5
Nucleophosmin (B23)	53.7
NOP10	39.1
Fibrillarin	33.6
NOP56	26.3
Dyskerin	24.3
NHP2	15.7
NHP2l1	6.3

## Data Availability

Data are contained within the article.
